# Virtual Reality in the Treatment of Adults with Chronic Low Back Pain: A Systematic Review and Meta-Analysis of Randomized Clinical Trials

**DOI:** 10.3390/ijerph182211806

**Published:** 2021-11-11

**Authors:** Beatriz Brea-Gómez, Irene Torres-Sánchez, Araceli Ortiz-Rubio, Andrés Calvache-Mateo, Irene Cabrera-Martos, Laura López-López, Marie Carmen Valenza

**Affiliations:** Physical Therapy Department, Faculty of Health Sciences, University of Granada, 18016 Granada, Spain; beatrizbreagomez@gmail.com (B.B.-G.); aortiz@ugr.es (A.O.-R.); andrescalvache@ugr.es (A.C.-M.); irenecm@ugr.es (I.C.-M.); lauralopez@ugr.es (L.L.-L.); cvalenza@ugr.es (M.C.V.)

**Keywords:** chronic low back pain, virtual reality, videogames, horse simulator riding, rehabilitation, physical therapy

## Abstract

Virtual reality (VR) can present advantages in the treatment of chronic low back pain. The objective of this systematic review and meta-analysis was to analyze the effectiveness of VR in chronic low back pain. This review was designed according to PRISMA and registered in PROSPERO (CRD42020222129). Four databases (PubMed, Cinahl, Scopus, Web of Science) were searched up to August 2021. Inclusion criteria were defined following PICOS recommendations. Methodological quality was assessed with the Downs and Black scale and the risk of bias with the Cochrane Risk of Bias Assessment Tool. Fourteen studies were included in the systematic review and eleven in the meta-analysis. Significant differences were found in favor of VR compared to no VR in pain intensity postintervention (11 trials; *n* = 569; SMD = −1.92; 95% CI = −2.73, −1.11; *p* < 0.00001) and followup (4 trials; *n* = 240; SDM = −6.34; 95% CI = −9.12, −3.56; *p* < 0.00001); and kinesiophobia postintervention (3 trials; *n* = 192; MD = −8.96; 95% CI = −17.52, −0.40; *p* = 0.04) and followup (2 trials; *n* = 149; MD = −12.04; 95% CI = −20.58, −3.49; *p* = 0.006). No significant differences were found in disability. In conclusion, VR can significantly reduce pain intensity and kinesiophobia in patients with chronic low back pain after the intervention and at followup. However, high heterogeneity exists and can influence the consistency of the results.

## 1. Introduction

Chronic low back pain (CLBP) is one of the main causes of pain, dysfunction, and disability [1,2]. It is one of the most common reasons for which patients require medical attention [3]. Furthermore, it is the world’s leading cause of years of life lived with disability [4]. In most cases, it is not possible to identify the specific nociceptive cause of CLBP and therefore, it is classified as nonspecific (pain not caused by a specific pathology such as infection, tumor, fracture, or inflammation) [2]. CLBP affects the physical, psychological, and social areas and carries a great socioeconomic burden, as it is the main cause of work absenteeism and the excessive use of therapeutic services [5]. For all these reasons, it is essential to establish an effective treatment.

There are many ways to treat CLBP in the clinical environment, such as surgery, medication, or physical therapy. In addition to analgesic treatment with drugs, manual therapy, pain management, and early physical exercise (coordination, strengthening, and resistance exercises) have been recommended with a strong level of evidence, as they can be beneficial in reducing pain and achieve a functional improvement [5,6]. However, in many cases the main limitation of physical exercise is lack of motivation and adherence [7]. Virtual reality (VR) can present some advantages in the face of these problems, since it contributes the motivational component and interactivity to the treatment [8]. The patient is involved in their recovery in a fun and attractive way and the interactive elements and feedback offered by the virtual environment can increase adherence to the exercises [9,10]. Negative thoughts and beliefs about pain experienced by some patients can lead to pain avoidance behaviors, causing inactivity, and preventing recovery and pain reduction [11]. VR treatment is a powerful pain distraction mechanism by focusing on an external stimulus and not on body movement, reducing attention to pain by dividing attention tasks [9,12]. Furthermore, compared to traditional methods, VR is considered a cost-effective and efficient tool [13].

In the current scientific literature, we found different reviews about VR in the treatment of pain in various areas. Gumaa et al. [14] explored VR effectiveness in orthopedic rehabilitation, showing inconclusive results in low back pain. In addition, they referred to the need for higher quality studies to establish more solid conclusions. In another review, VR in spinal pain was investigated [15]. Due to the low quality of the included studies, Ahern et al. [15] concluded that higher quality studies were necessary. A recent review published by Bordeleau et al. [16] concluded that while the specific set of studies showed high heterogeneity across several methodological factors, a tentative conclusion could be drawn that VR is effective improving back pain intensity and tends to have a positive effect on improving other pain outcomes and motion function. Bordeleau et al. [16] highlights that methodology framework for the development of VR treatments should be considered.

Since the completion of the search of the review of Bordeleau et al. [16], several new randomized clinical trials have been published on this topic, so there is new evidence to contribute to this issue. Additionally, a subgroup analysis of the different interventions is needed. Whether VR is applied alone or added to a physical therapy intervention could produce different results; furthermore, the comparison should also be taken into account.

Additionally, an analysis comparing the effects of the different VR interventions, the different durations of the interventions and the effects of VR at followup should be useful. It would also be of interest to explore other variables related to pain, in addition to pain intensity.

Therefore, the objective of this systematic review and meta-analysis of randomized clinical trials was to analyze the effectiveness of VR interventions in the treatment of CLBP. Implications and considerations may arise regarding the characteristics of the intervention programs.

## 2. Materials and Methods

### 2.1. Design

A systematic review was performed to identify randomized clinical trials exploring the effects of VR on the treatment of CLBP. The guidelines of the Preferred Reporting Items for Systematic Reviews and Meta-Analyses (PRISMA) [17] was used to carry out this systematic review. This systematic review was previously registered at the International prospective register of systematic reviews (PROSPERO) with number CRD42020222129. Available from: https://www.crd.york.ac.uk/prospero/display_record.php?RecordID=222129.

### 2.2. Search Strategy

Four databases were searched from their inception up to August 2021 without language restrictions. We used PubMed, Cinahl, Scopus, and Web of Science. The full search strategy is described in Appendix A. In order to find other relevant articles to the study, the reference list of other reviews and related articles were reviewed.

Additionally, a search was conducted for ongoing randomized clinical trials, which have not yet been published, to find out if they could be included in our review. The clinical trials registries ClinicalTrials.gov, the International Standard Randomized Controlled Trial Number (ISRCTN) Registry, and the International Clinical Trials Registry Platform (ICTRP) were used. Appendix B describes the search strategy used in each database.

### 2.3. Study Selection

The selection of studies was conducted systematically based on the prespecified PICOS (participants, interventions, comparisons, outcome, and study design) eligibility criteria: (1) Participants: adults (≥18 years) with CLBP (12 weeks or more) [18]; (2) Interventions: interventions based on VR; length of intervention of at least four weeks; (3) Comparisons: no intervention, interventions without VR, standard treatment, usual care, placebo or control; (4) Outcomes: pain intensity and other outcomes related to pain; (5) Study design: randomized clinical trials.

For the first screening title and abstract of each article was evaluated. We excluded those that did not meet the inclusion criteria defined with the PICOS strategy. After, the full text of relevant studies was assessed to check if they met the inclusion criteria. The list of excluded studies in the last screening and reason for exclusion is described in Appendix C.

When full text was not available, we contacted the corresponding author of the study via email. Two reviewers (BBG and ITS) independently carried out the search and selection of studies. If needed, disagreements were resolved with a third reviewer.

### 2.4. Data Extraction

The following data were recorded from the included articles: author, year of publication, country, disease, sample size, age (years), gender (percentage of males), outcome measures, main results (outcomes that showed significant differences (*p* ≤ 0.05)), measuring instrument, and time point assessment. This information is summarized in Table 1. In addition, the score obtained on the Downs and Black methodological quality scale [19] was added. Table 2 shows the characteristics of interventions: experimental group intervention, control group interventions, session duration, frequency, program duration, supervision, and adverse events.

When the information was insufficient or unclear, we contacted the corresponding author of the study via email. If the data were still unclear after contacting the corresponding author or if contact was not possible, it was analyzed using the available data. Two reviewers (BBG and ITS) independently carried out the data extraction. If needed, disagreements were resolved with a third reviewer.

### 2.5. Methodological Quality of Included Studies

Downs and Black quality assessment method [19] was used to assess the methodological quality of included studies in this review. This scale is one of the six best quality assessment scales [20,21,22]. This method contains 27 items divided into 5 sections: study quality (10 items), external validity (3 items), study bias (7 items), confounding and selection bias (6 items), and study power (1 item). In this review, we used the modified Downs and Black scale. The scoring for item 27 was simplified to a choice of 0 (“no”/“unable to determine”) or 1 point (“yes”). These scores will be the same for the rest of the items except item 5 which is valued as 0 (“no”/“unable to determine”), 1 (“partially”), or 2 (“yes”). Therefore, the scores range from 0 to 28 and the higher ones indicate a better methodological quality of the study [22,23]. According to their quality, studies can be categorized as excellent (26–28), good (20–25), fair (15–19), and poor (≤14) [22,23,24].

### 2.6. Risk of Bias of Included Studies

The Cochrane Risk of Bias Assessment Tool [25] was used to assess the risk of bias of included studies. This tool assesses seven domains: random sequence generation, allocation concealment, blinding of participants and personnel, blinding of outcome assessment, incomplete outcome data, selective reporting, and other bias. For each study, the different domains were scored as “high risk of bias”, “low risk of bias”, or “unclear”.

Two reviewers (BBG and ITS) carried out the assessment of risk of bias, as well as the assessment of methodological quality independently, and in case of doubt or disagreement a third reviewer was consulted.

### 2.7. Statistical Analysis

We used the Review Manager (RevMan) software version 5.4 to perform statistical analysis and used forest plots to display the results. Analysis was performed for those outcomes repeated at least in three comparisons or studies. Regarding the period of time, the analysis was carried out after the intervention and at 6 months followup. Mean, standard deviation (SD) and sample size were extracted from included studies to estimate the overall effect. For continuous outcomes, the mean difference (MD) and the 95% confidence intervals (CI) were used when the outcomes were evaluated with the same scale and the standardized mean difference (SMD) when the scales were different. The method utilized was inverse variance. The fixed effects model was used and the random effects model was applied when heterogeneity was greater than 75%. A value of *p* ≤ 0.05 was considered statistically significant. Heterogeneity between studies was assessed using the I^2^ test. The degree of heterogeneity was categorized as low (I^2^ < 25%), moderate (I^2^ = 25–75%), and high (I^2^ > 75%). In order to explore possible causes of heterogeneity among study results we conducted a subgroup analysis. Subgroups were performed according to the comparisons (VR vs. no intervention, VR vs. placebo, VR vs. oral treatment, VR vs. physiotherapy, VR + physiotherapy vs. physiotherapy, and VR + physiotherapy vs. no VR exercise + physiotherapy); the type of intervention with VR (Nintendo consoles, Horse Simulator Riding, and Prokin System), and the duration of the intervention (4, 8, or 12 weeks).

## 3. Results

### 3.1. Search Selection

After the initial search in the databases and reference lists, we found 1363 manuscripts. After removing duplicates, we obtained 838 potentially eligible records. After screening by title and abstract, 58 articles remained, of which the full text was assessed. Of those 58 studies, 14 randomized clinical trials met the inclusion criteria, and finally 11 were included in quantitative synthesis.

**Figure 1:**
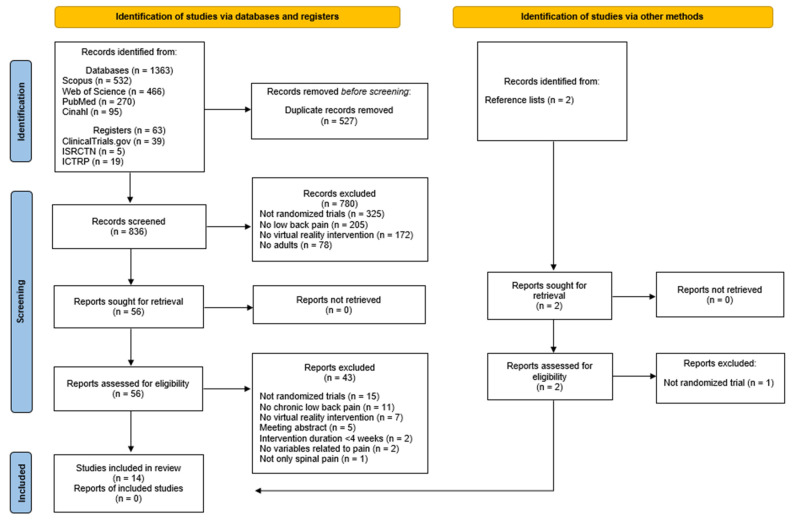
PRISMA flow diagram: database and clinical trials register search and other sources.

In addition, we searched for ongoing randomized clinical trials. Of the 63 studies found in the three clinical trial registries consulted, 17 finally met the inclusion criteria. Figure 1 shows the flow diagram of the articles during the study selection process in the databases and clinical trial registries. The list of ongoing randomized clinical trials that could be included in the review is shown in Appendix D. None of the ongoing randomized clinical trials were included in this review.

### 3.2. Characteristics of Included Studies

Table 1 shows the characteristics of included studies in this review. All studies were randomized clinical trials and are arranged chronologically from oldest to newest. The included studies were published between 2013 and 2021.

Six studies were conducted in South Korea [26,27,28,29,30,31], one in Brazil [32], one in the USA [33], one in Australia [7], three in Saudi Arabia [34,35,36], one in Turkey [37], and one in Japan [38]. The total number of participants was 765. The mean age of the participants was 40.04 with 62.08% men. All studies measured pain intensity. It was measured using VAS in nine studies [26,27,28,29,31,34,35,36,38], 11-point Numeric Pain Rating Scale (11-NPRS) in four studies [7,30,32,37], and The Defense and Veterans Pain Rating Scale (DVPRS) in one study [33]. Four studies measured disability associated with low back pain using the Oswestry Disability Index (ODI) [29,30,31,37], four studies measured kinesiophobia using the 17-item Tampa Scale of Kinesiophobia (17-TSK) [7,35,36,38], and four studies measured body composition using bioelectrical impedance analysis method [27,28,31,38]. Other variables assessed more frequently were severity of disability with Roland Morris Disability Questionnaire (RMDQ) in two studies [7,30], isokinetic trunk flexion/extension with a dynamometer in three studies [27,28,31], pain self-efficacy with the 10-item Pain Self-Efficacy (10-PSEQ) in two studies [7,38], pain catastrophizing with Pain Catastrophizing Scale (PCS) in two studies [33,38], and blood serum levels of stress hormones in two studies [35,36]. Variables were assessed before and after the intervention in all articles. Five studies included followup, one at 8 weeks and 6 months [34], other at 3 and 6 months [7], and three only at 6 months [30,35,36]. In addition, one of these studies included a midterm assessment after 4 weeks [30], and one study assessed outcomes during intervention [33].

**Table 1:** Characteristics of included studies.

Author (Year) [Ref.]	Country	Sample Size	Age (Years) Mean ± SD	Gender (% Males)	Outcome Measures	Measuring Instrument	Time Points Assessment	Quality
Park et al. (2013) [26]	South Korea	n = 24 EG1:8 EG2: 8 CG: 8	EG1: 44.12 ± 5.48 EG2: 43.37 ± 5.42 CG: 45.50 ± 5.34	100	Pain intensity Back strength Functional balance Health status	VAS Isometric lifting strength One-legged stand test RAND-36	Pre-intervention Post-intervention	16
Oh et al. (2014) [27]	South Korea	n = 37 EG1: 10 EG2: 9 EG3: 9 CG: 9	EG1: 20.56 ± 0.69 EG2: 20.33 ± 0.52 EG3: 20.44 ± 0.27 CG: 20.70 ± 0.37	100	**Pain intensity** Body composition **Isokinetic trunk flexion/extension** **Isokinetic hip flexion/extension and ABD/ADD**	VAS Bioelectrical impedance analysis method and anthropometer Isokinetic dynamometer Isokinetic dynamometer	Pre-intervention Post-intervention	15
Yoo et al. (2014) [28]	South Korea	n = 47 EG: 24 CG: 23	EG: 20.44 ± 1.33 CG: 20.70 ± 1.45	100	**Pain intensity** **Body composition** ** ** **Isokinetic trunk flexion/extension**	VAS Bioelectrical impedance analysis method Isokinetic dynamometer	Pre-intervention Post-intervention	17
Monteiro-Junior et al. (2015) [32]	Brazil	n = 30 EG: 16 CG: 14	68 ± 4	0	Pain intensity Static balance Functional capacity Mood	11-NPRS Wii balance board Sit-to-stand test POMS	Pre-intervention Post-intervention	21
Chen et al. (2016) [29]	South Korea	n = 19 EG: 10 CG: 9	19-30	-	Pain intensity Disability associated with low back pain Dynamic balance	VAS KODI LoS test with Biorescue	Pre-intervention Post-intervention	13
Zadro et al. (2019) [7]	Australia	n = 60 EG: 30 CG: 30	EG: 68.8 ± 5.5 CG: 67.8 ± 6	EG: 20 CG: 28.3	**Pain self-efficacy** Care-seeking **Physical activity** Pain intensity **Function changes** Severity of disability Kinesiophobia Falls efficacy	10-PSEQ 3-items questionnaire The Rapid Assessment of Physical Activity Questionnaire 11-NPRS PSFS RMDQ 17-TSK FEQ-I	Pre-intervention Post-intervention 3 months follow-up 6 months follow-up	26
Kim et al. (2020) [30]	South Korea	n = 48 EG: 24 CG: 24	EG: 26 ± 3.82 CG: 28.79 ± 9.05	EG: 68.2 CG: 42.3	Pain intensity Disability associated with low back pain Severity of disability **Fear and avoidance beliefs**	11-NPRS ODI RMDQ FABQ	Pre-intervention 4 weeks Post-intervention 6 months follow-up	22
Nambi et al. (2020) A [34]	Saudi Arabia	n = 45 EG1: 15 EG2: 15 CG: 15	EG1: 21.25 ± 1.2 EG2: 20.23 ± 1.6 CG: 20.78 ± 1.6	100	**Pain intensity** **Player wellness** **Sprint performance:** **- 40 m sprint and 4 × 5 m sprint** **- Submaximal shuttle running** **Jump performance: CJ and SJ**	VAS Player wellness questionnaire Photocell timer MEMS device Optical timing system	Pre-intervention Post-intervention 8 weeks follow-up 6 months follow-up	25
Nambi et al. (2020) B [35]	Saudi Arabia	n = 60 EG1: 20 EG2:20 CG:20	EG1: 23.2 ± 1.5 EG2: 22.8 ± 1.6 CG: 23.3 ± 1.5	100	**Pain intensity** **Kinesiophobia** **Blood serum levels of stress hormones**	VAS 17-TSK 20 ml venous blood sample	Pre-intervention Post-intervention 6 months follow-up	24
Park et al. (2020) [31]	South Korea	n = 80 EG: 40 CG:40	EG: 71.50 ± 6.34 CG: 72.05 ± 6.82	0	**Pain intensity** **Disability associated with low back pain** **Isokinetic trunk flexion/extension** **Body composition** ** ** **Spinal alignment**	VAS ODI Isokinetic dynamometer Bioelectrical impedance analysis method Raster stereography	Pre-intervention Post-intervention	21
Tomruk et al. (2020) [37]	Turkey	n = 42 EG: 21 CG: 21	Median (IQR) EG: 46 (40.05-50.50) CG: 45 (44-48)	-	**Pain intensity** **Disability associated with low back pain** **Postural control** Physical activity	11-NPRS ODI LoS PS tests by Biodex Balance System SenseWear Armband	Pre-intervention Post-intervention	18
García et al. (2021) [33]	USA	n = 179 EG: 89 CG: 90	EG: 51.5 ± 13.5 CG: 51.4 ± 12.9	EG: 25 CG: 21	**Pain intensity** **Pain interference** Patient’s global impression of change **Physical function and sleep disturbances** Pain catastrophizing Pain self-efficacy Chronic pain acceptance Satisfaction with treatment VR device use System usability Over-the-counter analgesic medication use Opioid use data	DVPRS DVPRS-II Question and 7-point scale ranging PROMIS PCS PSEQ-2 CPAG-8 6-point scale Device SUS Question yes/no Self-reported	Pre-intervention During intervention Post-intervention	27
Nambi et al. (2021) [36]	Saudi Arabia	n = 54 EG1: 18 EG2:18 CG:18	EG1: 22.3 ± 1.6 EG2: 21.4 ± 1.8 CG: 21.9 ± 1.8	100	**Pain intensity** **Kinesiophobia** **Blood serum levels of hormones**	VAS 17-TSK 20 ml venous blood sample	Pre-intervention Post-intervention 6 months follow-up	24
Sato et al. (2021) [38]	Japan	n = 40 EG: 20 CG: 20	EG: 49.31 ± 12.59 CG: 55.61 ± 10.96	EG: 45 CG: 40	**Pain intensity** **Buttock pain** Leg numbness Body composition Left and right GS **Pain self-efficacy** Pain catastrophizing Kinesiophobia	VAS VAS VAS Bioelectrical impedance analysis method Dynamometer 10-PSEQ PCS 17-TSK	Pre-intervention Post-intervention	22

SD: Standard Deviation; EG: experimental group; CG: control group; LBP: Low Back Pain; VAS: Visual Analogue Scale; RAND-36: RAND-36 Health Status Inventory; CLBP: Chronic Low Back Pain; ABD: Abduction; ADD: adduction; 11-NPRS: Numeric Pain Rating Scale; POMS: Profile of Mood States; KODI: the Korean Oswestry Disability Index; LoS: Limits of Stability; 10-PSEQ: 10 items Pain-Self Efficacy; Questionnaire; PSFS: Patients Specific Functional Scale; RMSQ: Roland Morris Disability Questionnaire;17-TSK: 17 item Tampa Scale of Kinesiophobia; FEQ-I: Falls Efficacy Scale Questionnaire International, ODI: Oswestry Disability Index; FABQ: Fear-avoidance Beliefs Questionnaire; CJ: Countermovement jump, SJ: Squat jump; PS: Postural Stability; DVPRS: The Defense and Veterans Pain Rating Scale; DVPRS-II: The Defense and Veterans Pain Rating Scale interference; PROMIS: The NIH Physical Function and Sleep Disturbance; PCS: Pain Catastrophizing Scale; PSEQ-2: 2 items Pain-Self Efficacy Questionnaire; CPAG8: 8 item Chronic Pain Acceptance Questionnaire; SUS: System Usability Scale; GS: Grip Strength. Outcomes with significant differences (*p* < 0.05) between groups are presented in bold.

### 3.3. Characteristics of Interventions

Characteristics of the interventions of the included studies are described in Table 2.

Regarding the interventions, three studies compared VR with no intervention [7,27,28], two studies with a placebo [31,33], and other study with oral treatment (Nonsteroidal Anti-Inflammatory Drugs (NSAIDs), tramadol, and duloxetine) [38]. In two studies, comparisons consisted of VR versus physiotherapy [30,37]. In addition, three studies combined VR + physiotherapy versus physiotherapy alone [26,29,32], and four studies combined those interventions and compared them with no VR exercises and physiotherapy [26,34,35,36].

Four of the fourteen studies used Nintendo programs and consoles [7,26,32,38], whereas three studies used other types of video games with sensors and a monitor (Prokin System) [34,35,36]. Five studies used a horse simulator riding [27,28,29,30,31]. One study used a system similar to VR, but without video games, based on biofeedback [37], and in one study, the intervention was a behavioral skills-based VR program with VR glasses [33].

The mean time using VR was 28.29 min and the mean session duration was 46.21 min. Regarding the frequency of the sessions, it varied from one weekly session [38] to seven sessions per week [33]. The duration of the program in the different studies ranged from 4 [29,34,35,36] to 12 weeks [31,37]. In nine studies, the interventions were supervised [27,28,30,31,32,34,35,36,37]. In one article, participants were contacted by phone calls [7], and one did not include any type of supervision [33]. Three studies did not report on supervision of the intervention [26,29,38]. Of all the articles, only two reported adverse events derived from the intervention with VR (e.g., nausea, motion sickness, vertigo, etc.) [32,33]; in two articles no adverse events were reported [7,30], and in the rest no information was provided.

### 3.4. Methodological Quality of Included Studies

Downs and Black quality assessment method [19] was used to assess the methodological quality of included studies in this review. The total score for each study is shown in Table 1, and the score for each item is summarized in Appendix E. According to their score, of the 14 articles evaluated, two were classified as excellent (26–28), seven as good (20–25), four as fair (19–15), and one as poor (≤14). The mean score of the included studies was 20.79 (range: 13–27).

### 3.5. Risk of Bias of Included Studies

**Figure 2:**
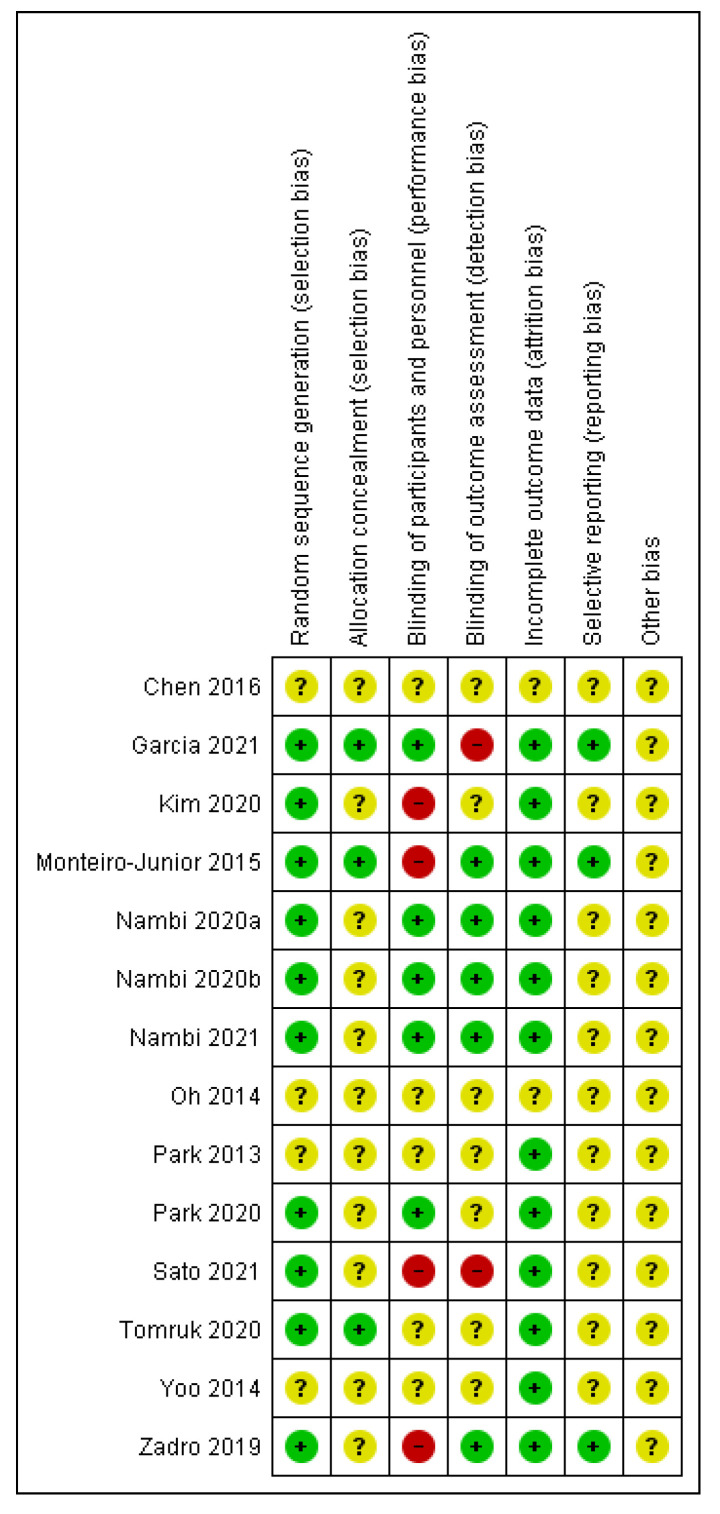
Risk of bias summary.

**Figure 3:**
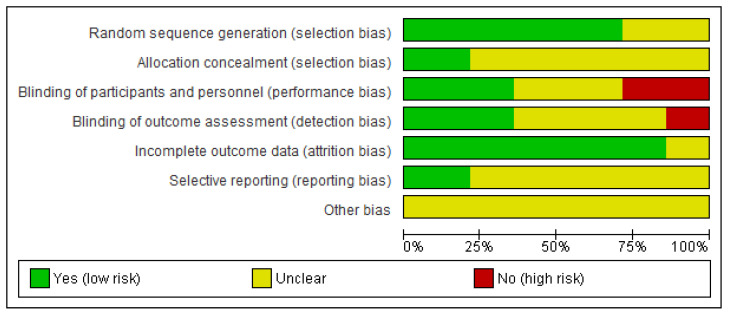
Risk of bias graph.

The Cochrane Risk of Bias Assessment Tool [25] was used to assess the risk of bias of the articles included in this review. Figure 2 and Figure 3 show the summary and the graph of the risk of bias assessment, respectively. Random sequence generation, allocation concealment, incomplete outcome data, and selective reporting did not obtain a high risk of bias in any study. In addition, other bias obtained unclear risk of bias in all of the included studies. Blinding of participants and personnel and blinding of outcome assessment was evaluated as a high risk of bias in four [7,30,32,38] and two [33,38] studies, respectively. Two studies obtained unclear risk of bias in all items [29,37] and other two studies obtained unclear risk of bias in all items, except in incomplete outcome data [26,28].

### 3.6. Effects of Virtual Reality vs. No Virtual Reality in Chronic Low Back Pain

For meta-analysis, we only considered the outcome pain intensity and outcomes related to pain.

Eleven studies were included in the meta-analysis. All of them were included for pain intensity postintervention; four for pain intensity at the 6 month followup; three for disability postintervention; three for kinesiophobia postintervention, and two (four comparisons) for kinesiophobia at the six months followup. Two articles were excluded from the meta-analysis because they did not express data in mean ± SD [33,37]. In addition, Yoo et al. [28] was excluded because the SD was 0, and it was not estimable by RevMan.

#### 3.6.1. Subgroup Based on Intervention Comparisons: Virtual Reality Alone or Combined with Physiotherapy vs. Control Group Interventions

Firstly, a subgroup analysis of the different interventions was performed to know if VR applied alone or added to a physical therapy intervention could produce different results, and if it differed depending on the type of intervention of the control group. We analyzed pain intensity, disability, and kinesiophobia postintervention; and pain intensity and kinesiophobia at the 6 months followup. The Visual Analog Scale (VAS) to evaluate pain intensity was adjusted to a scale of 0–10 cm when it was expressed in millimeters.

**Figure 4:**
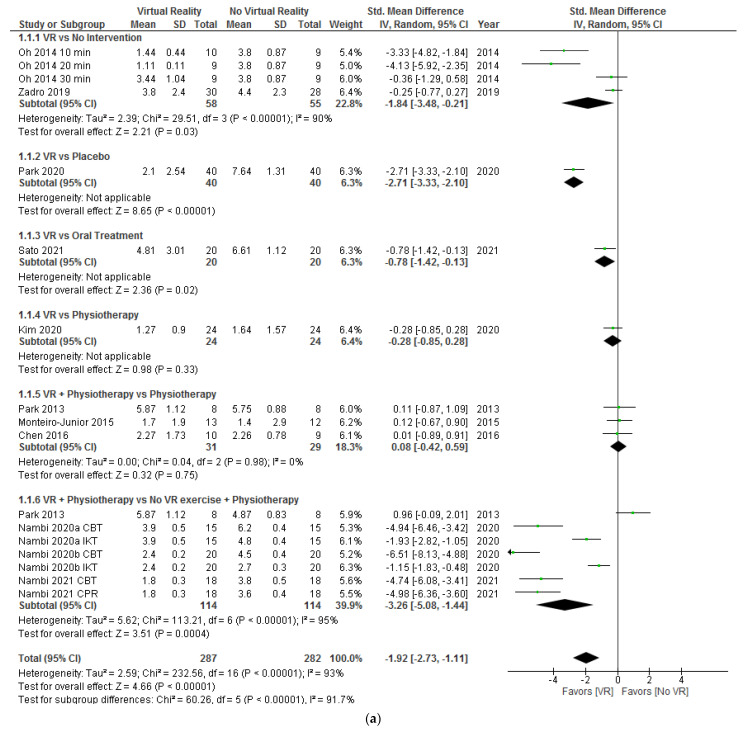

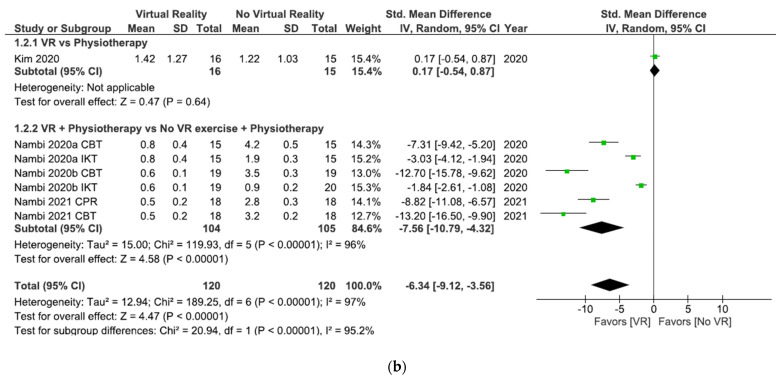
Effect of virtual reality versus no virtual reality in chronic low back pain for pain intensity postintervention (**a**) and at the six month followup (**b**) based on the type of intervention. CBT: conventional balance training; IKT: isokinetic training; CPR: combined physical rehabilitation; VR: virtual reality.

In Figure 4a, the results show significant differences (SMD = −1.92; 95% CI = −2.73, −1.11; *p* < 0.00001) in favor of VR compared to no VR in pain intensity postintervention. When VR was compared with no intervention (SMD = −1.84; 95% CI = −3.48, −0.21; *p* = 0.03), placebo (SMD = −2.71; 95% CI = −3.33, −2.10; *p* < 0.00001), or oral treatment (SMD = −0.78; 95% CI = −1.42, −0.13; *p* = 0.02), the subgroup analysis showed significant differences in favor of VR. In addition, when VR + physiotherapy were compared with no VR exercise + physiotherapy, the subgroup analysis showed significant differences (SMD = −3.26; 95% CI = −5.08. −1.44; *p* = 0.0004) in favor of VR too. However, no significant differences were observed between VR and physiotherapy (SMD = −0.28; 95% CI = −0.85, 0.28; *p* = 0.33) or VR + physiotherapy and physiotherapy (SMD = 0.08; 95% CI = −0.42, 0.59; *p* = 0.75). Heterogeneity was high in overall effect (I^2^ = 93%; *p* < 0.00001) and in two subgroups, VR versus no intervention (I^2^ = 90%; *p* < 0.00001) and VR + physiotherapy versus no VR exercise + physiotherapy (I^2^ = 95%; *p* < 0.00001). According to the I^2^ statistic, 0% of variation across studies was due to heterogeneity (*p* = 0.98) in VR + physiotherapy versus the physiotherapy subgroup.

In Figure 4b, the results show significant differences (SDM = −6.34; 95% CI = −9.12, –3.56; *p* < 0.00001) in pain intensity at the six month followup in favor of VR compared to no VR. When VR was compared with physiotherapy, the subgroup analysis showed no significant differences (SDM = 0.17; 95% CI = −0.54, 0.87; *p* = 0.64). However, when VR + physiotherapy were compared with no VR exercise + physiotherapy, the subgroup analysis showed significant differences in favor of VR (SDM = −7.56; 95% CI = −10.79, –4.32; *p* < 0.00001). Heterogeneity was high in overall effect (I^2^ = 97%; *p* < 0.00001) and in VR + physiotherapy versus no VR exercise + physiotherapy subgroup (I^2^ = 96%; *p* < 0.00001).

**Figure 5:**
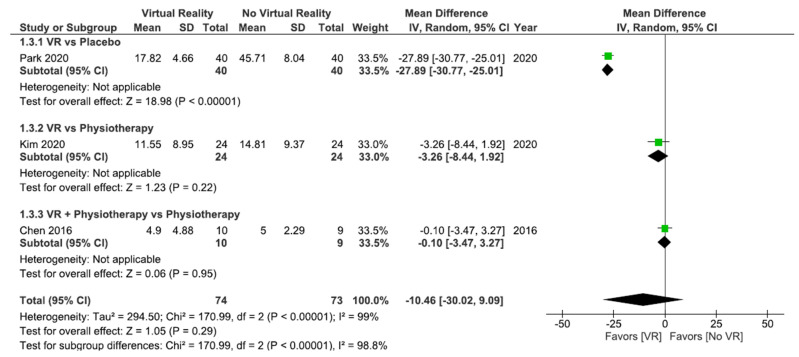
Effect of virtual reality versus no virtual reality in chronic low back pain for disability postintervention based on the type of intervention. VR: virtual reality.

As shown in Figure 5, no significant differences were found between VR interventions and other interventions without VR (MD = 10.46; 95% CI = −30.02, 9.09; *p* = 0.29) in disability postintervention. Subgroup analysis did not show significant differences between VR and physiotherapy (MD = −3.26; 95% CI = −8.44, 1.92; *p* = 0.22) or between VR + physiotherapy and physiotherapy (MD = −0.10; 95% CI = −3.47, 3.27; *p* = 0.95). However, when VR was compared with the placebo, the subgroup analysis showed significant differences in favor of VR (MD = −27.89; 95% CI = −30.77, –25.01; *p* < 0.00001). Heterogeneity between studies was high (I^2^ = 99%; *p* < 0.00001).

**Figure 6:**
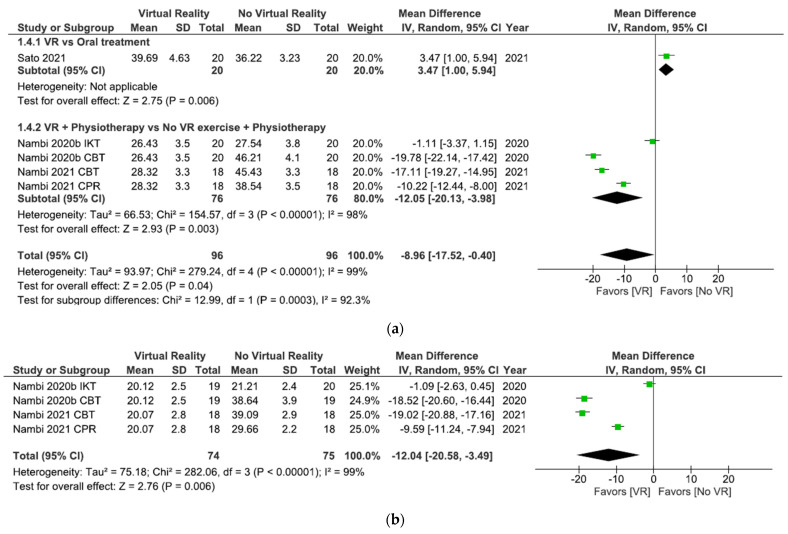
Effect of virtual reality versus no virtual reality in chronic low back pain for kinesiophobia postintervention (**a**) and at the six month followup (**b**) based on the type of intervention. IKT: isokinetic training; CBT: conventional balance training; CPR: combined physical rehabilitation; VR: virtual reality.

As shown in Figure 6a, the results showed significant differences (MD = −8.96; 95% CI = −17.52, –0.40; *p* = 0.04) in favor of VR in total comparison in kinesiophobia postintervention. When VR was compared with oral treatment, the subgroup analysis showed significant differences in favor of oral treatment (MD = 3.47; 95% CI = 1.00, 5.94; *p* = 0.006). However, when VR + physiotherapy were compared with no VR exercises + physiotherapy, the subgroup analysis showed significant differences in favor of VR (MD = −12.05; 95% CI = −20.13, –3.98; *p* = 0.003). Heterogeneity was high in overall effect (I^2^ = 99%; *p* < 0.00001) and in VR + physiotherapy versus no VR exercise + physiotherapy subgroup (I^2^ = 98%; *p* < 0.00001).

All studies in this meta-analysis (Figure 6b) compared VR + physiotherapy versus no VR exercise + physiotherapy. The results showed significant differences (MD = −12.04; 95% CI = −20.58, –3.49; *p* = 0.006) in favor of VR in kinesiophobia at the 6 month followup. Heterogeneity between studies was high (I^2^ = 99% *p* < 0.00001).

#### 3.6.2. Subgroups Based on Virtual Reality Interventions

Other subgroup analysis was based on the type of VR intervention. The studies were divided into three subgroups: Nintendo consoles, Horse Simulator Riding, or Prokin System. We analyzed pain intensity, disability, and kinesiophobia postintervention and pain intensity and kinesiophobia at the 6 months followup.

**Figure 7:**
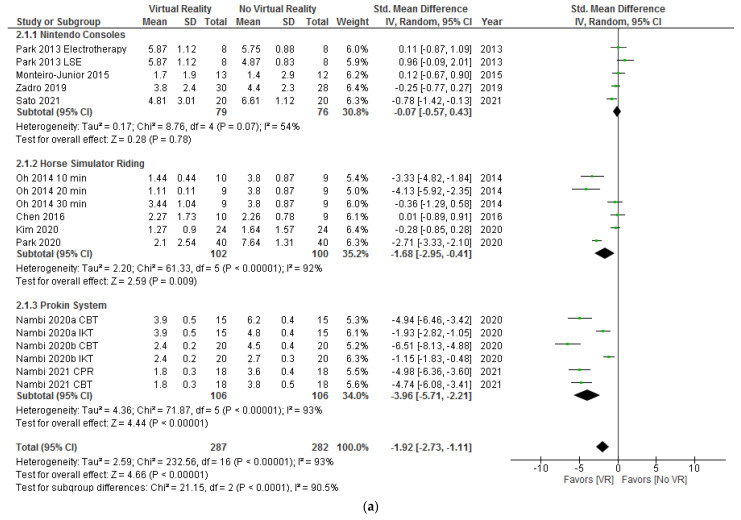

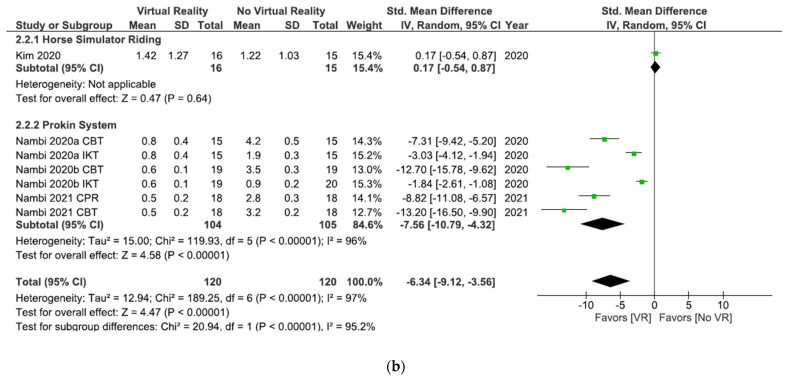
Effect of virtual reality versus no virtual reality in chronic low back pain for pain intensity postintervention (**a**) and at the six months followup (**b**) based on the type of virtual reality intervention. CBT: conventional balance training; IKT: isokinetic training; CPR: combined physical rehabilitation; VR: virtual reality.

As shown in Figure 7a, the results showed significant differences (SMD = −1.92; 95% CI = −2.73, −1.11; *p* < 0.00001) in favor of VR versus no VR in pain intensity postintervention. When Nintendo consoles were compared with interventions without VR, the subgroup analysis showed no significant differences (SMD = −0.07; 95% CI = −0.57, 0.43; *p* = 0.78). However, when horse simulator riding (SMD = −1.68; 95% CI = −2.95, –0.41; *p* = 0.009) or Prokin System (SMD = −3.96; 95% CI = −5.71, –2.21; *p* < 0.00001) were compared with interventions without VR, the subgroup analysis showed significant differences in favor of VR. Heterogeneity was high in overall effect (I^2^ = 93%; *p* < 0.00001) and in two subgroups, horse simulator riding (I^2^ = 92%; *p* < 0.00001) and Prokin System (I^2^ = 93%; *p* < 0.00001). According to the I^2^ statistic, 54% of variation across studies was due to heterogeneity (*p* = 0.07) in the Nintendo consoles subgroup.

As shown in Figure 7b the results showed significant differences (SDM = −6.34; 95% CI = −9.12, –3.56; *p* < 0.00001) in favor of VR in total comparison in pain intensity at the 6 month followup. Regarding subgroup analysis, no significant differences were found between horse simulator riding and no VR interventions (SDM = 0.17; 95% CI = −0.54, 0.87; *p* = 0.64). However, significant differences in favor of VR were found in the Prokin System subgroup (SDM = −7.56; 95% CI = −10.79, –4.32; *p* < 0.00001). Heterogeneity was high in overall effect (I^2^ = 97%; *p* < 0.00001) and in Prokin System versus no VR interventions subgroup (I^2^ = 96%; *p* < 0.00001).

**Figure 8:**
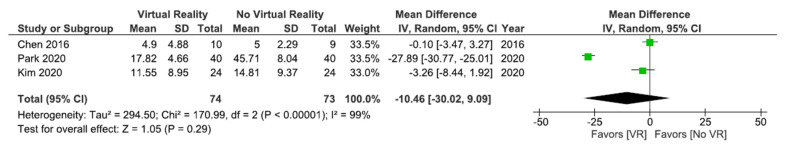
Effect of virtual reality versus no virtual reality in chronic low back pain for disability postintervention based on the type of virtual reality intervention. VR: virtual reality.

All studies in Figure 8 compared horse simulator riding interventions versus other interventions without VR. No significant differences were found between VR and no VR (MD = −10.46; 95% CI = −30.02, 9.09; *p* = 0.29) in disability post-intervention. Heterogeneity between studies was high (I^2^ = 99%; *p* < 0.00001).

**Figure 9:**
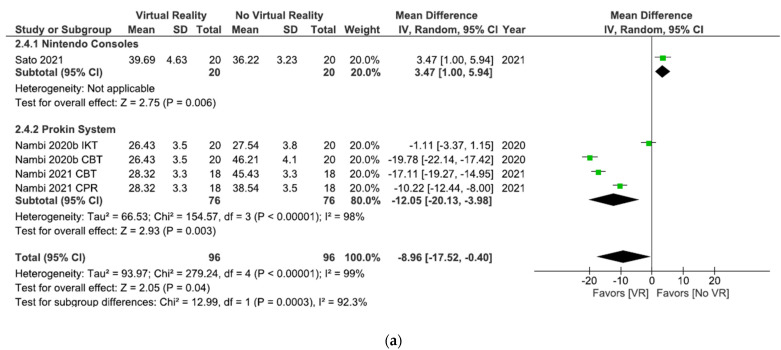

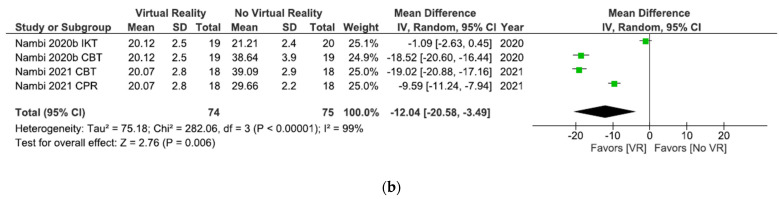
Effect of virtual reality versus no virtual reality in chronic low back pain for kinesiophobia postintervention (**a**) and at the six month followup (**b**) based on the type of virtual reality intervention. IKT: isokinetic training; CBT: conventional balance training; CPR: combined physical rehabilitation; VR: virtual reality.

As shown in Figure 9a, the results showed significant differences (MD = −8.96; 95% CI = −17.52, −0.40; *p* = 0.04) in favor of VR in total comparison in kinesiophobia postintervention. The results showed significant differences in favor of interventions without VR versus interventions with Nintendo consoles (MD = 3.47; 95% CI = 1.00, 5.94; *p* = 0.006). However, when the Prokin System was compared with interventions without VR significant differences were found in favor of the Prokin System subgroup (MD = −12.05; 95% CI = −20.13, −3.98; *p* = 0.003). Heterogeneity was high in overall effect (I^2^ = 99%; *p* < 0.00001) and in Prokin System versus interventions without VR (I^2^ = 98%; *p* < 0.00001).

All studies in Figure 9b compared Prokin System versus interventions without VR. The results showed significant differences (MD = −12.04; 95% CI = −20.58, −3.49; *p* = 0.006) in favor of VR in kinesiophobia at the 6 month followup. Heterogeneity between studies was high (I^2^ = 99%; *p* < 0.00001).

#### 3.6.3. Subgroups Based on the Duration of the Intervention

The last subgroup analysis was based on the duration of the intervention. The studies were divided into three subgroups: four weeks, eight weeks, or twelve weeks of intervention. We analyzed pain intensity, disability, and kinesiophobia postintervention and pain intensity and kinesiophobia at the 6 month followup.

**Figure 10:**
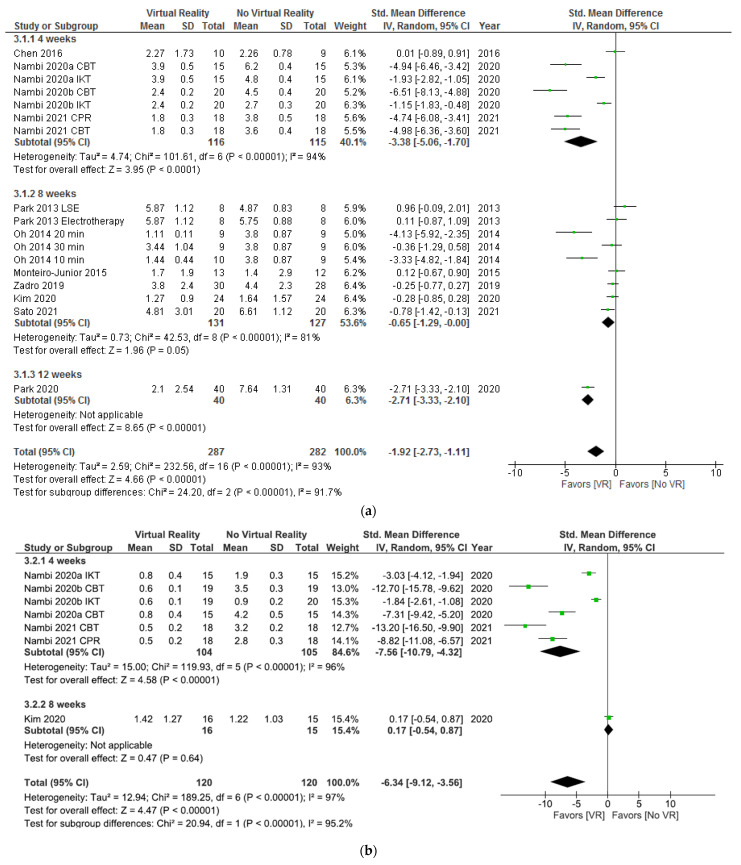
Effect of virtual reality versus no virtual reality in chronic low back pain for pain intensity postintervention (**a**) and at the six month followup (**b**) based on the duration of the intervention. CBT: conventional balance training; IKT: isokinetic training; CPR: combined physical rehabilitation; VR: virtual reality.

As shown in Figure 10a, the results showed significant differences (SMD = −1.92; 95% CI = −2.73, −1.11; *p* < 0.00001) in favor of VR versus no VR in pain intensity postintervention. Subgroup analysis showed significant differences in favor of VR after 4 weeks of intervention (SMD = −3.38; 95% CI = −5.06, −1.70; *p* < 0.0001), 8 weeks of intervention (SMD = −0.65; 95% CI = −1.29, −0.00; *p* = 0.05), and 12 weeks of intervention (SMD = −2.71; 95% CI = −3.33, −2.10; *p* < 0.00001). Heterogeneity was high in overall effect (I^2^ = 93%; *p* < 0.00001) and in all subgroups (I^2^ = 94%; *p* < 0.00001) (I^2^ = 81%; *p* < 0.00001).

As shown in Figure 10b, the results showed significant differences (SDM = −6.34; 95% CI = −9.12, −3.56; *p* < 0.00001) in favor of VR in total comparison in pain intensity at the 6 month followup. Regarding subgroup analysis, no significant differences were found between VR versus no VR after 8 weeks of intervention (SDM = 0.17; 95% CI = −0.54, 0.87; *p* = 0.64). However, significant differences in favor of VR were found after 4 weeks of intervention (SDM = −7.56; 95% CI = −10.79, −4.32; *p* < 0.00001). Heterogeneity was high in overall effect (I^2^ = 97%; *p* < 0.00001) and in the 4 weeks of intervention subgroup (I^2^ = 96%; *p* < 0.00001).

**Figure 11:**
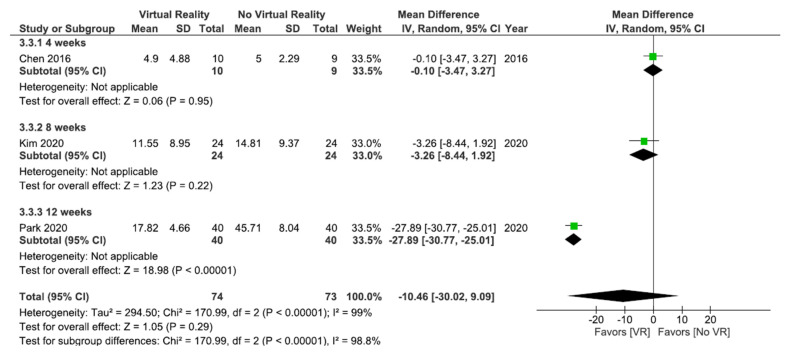
Effect of virtual reality versus no virtual reality in chronic low back pain for disability postintervention based on the duration of the intervention. VR: virtual reality.

No significant differences were found between VR interventions and other interventions without VR (MD = −10.46; 95% CI = −30.02, 9.09; *p* = 0.29) in disability postintervention. Subgroup analysis did not show significant differences between VR and no VR after 4 weeks (MD = −0.10; 95% CI = −3.47, 3.27; *p* = 0.95) or 8 weeks of intervention (MD = −3.26; 95% CI = −8.44, 1.92; *p* = 0.22). However, significant differences were found in favor of VR after 12 weeks of intervention (MD = −27.89; 95% CI = −30.77, –25.01; *p* < 0.00001). Heterogeneity between studies was high (I^2^ = 99%; *p* < 0.00001). Figure 11 shows these results.

**Figure 12:**
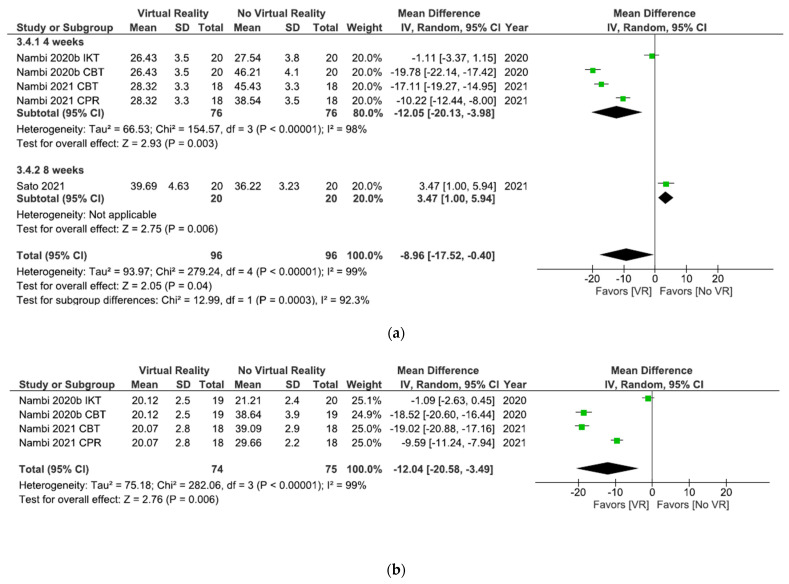
Effect of virtual reality versus no virtual reality in chronic low back pain for kinesiophobia postintervention (**a**) and at the six month followup (**b**) based on the duration of the intervention. IKT: isokinetic training; CBT: conventional balance training; CPR: combined physical rehabilitation; VR: virtual reality.

As shown in Figure 12a, the results showed significant differences (MD = −8.96; 95% CI = −17.52, −0.40; *p* = 0.04) in favor of VR in total comparison in kinesiophobia postintervention. After 8 weeks of intervention, the results showed significant differences in favor of no VR intervention (MD = 3.47; 95% CI = 1.00, 5.94; *p* = 0.006). However, significant differences in favor of VR were observed after 4 weeks of intervention (MD = −12.05; 95% CI = −20.13, −3.98; *p* = 0.003). Heterogeneity was high in overall effect (I^2^ = 99%; *p* < 0.00001) and in the 4 weeks of intervention subgroup (I^2^ = 98%; *p* < 0.00001).

All studies shown in Figure 12b conducted a 4-week intervention. The results showed significant differences (MD = −12.04; 95% CI = −20.58, –3.49; *p* = 0.006) in favor of VR in kinesiophobia at the 6 month followup. Heterogeneity between studies was high (I^2^ = 99%; *p* < 0.00001).

## 4. Discussion

The objective of this systematic review and meta-analysis was to analyze the effectiveness of VR interventions in the treatment of CLBP. Fourteen studies were included in this review and eleven of them in the meta-analysis. The results showed significant differences in favor of VR interventions in pain intensity and kinesiophobia postintervention and at the six month followup. However, no significant differences were found in disability postintervention.

### 4.1. Pain Intensity

Pain intensity was assessed in all of the studies included in meta-analysis. The meta-analysis showed significant differences in favor of interventions with VR versus interventions without VR in pain intensity postintervention and at the six month followup. On the one hand, the effect of VR was superior to no intervention [7,27], placebo [31], and oral treatment [38] in pain intensity postintervention, but it should be noted that there was only a study in two of these subgroups. Significant differences in favor of VR + physiotherapy were observed when we compared with no VR exercise + physiotherapy [26,34,35,36] in pain intensity postintervention and at the six month follow-up. Most of the studies included in this subgroup had good methodological quality and obtained significant differences in favor of VR in the rest of the variables not included in the meta-analysis. However, it must be taken into account that these results have been obtained from studies that only included young athletic men and cannot be generalized [34,35,36]. On the other hand, VR was not superior to physiotherapy in pain intensity postintervention or at the six month followup. Nevertheless, there was only one article (with young adults and a high dropout rate) in this subgroup [30]. Neither were significant differences found between VR + physiotherapy versus physiotherapy in pain intensity postintervention. It should be noted that these studies had a small sample size and some of them had low methodological quality [26,29,32].

Regarding the type of VR, horse simulator riding and Prokin System were superior to interventions without VR in pain intensity postintervention. However, in the horse simulator riding subgroup, most of the studies compared this type of VR with no intervention [27] or placebo [31], which can explain the good results in the analysis. Nintendo consoles did not show significant differences. This may be because the Prokin System and horse simulator riding are specialized VR devices compared to Nintendo consoles. At the six month followup, the results showed significant differences in favor of the Prokin System but not in favor of horse simulator riding. It must be taken into account that there was only one study (with young adults and a high dropout rate) in this subgroup [30]. The results showed significant differences between VR and no VR in pain intensity postintervention after 4 weeks, 8 weeks, or 12 weeks of intervention. At the six month followup, significant differences in favor of VR were found after 4 weeks of intervention but not after 8 weeks of intervention. It should be noted that there was only one study in this subgroup [30].

### 4.2. Disability

No significant differences were found between VR interventions (horse simulator riding) and no VR interventions in disability postintervention. However, when VR was compared with placebo and when the intervention lasted 12 weeks, the different subgroups analysis showed significant differences in favor of VR. This can be explained because the same article was included in the subgroups [31]. No significant differences were observed between VR and physiotherapy [30] or VR + physiotherapy and physiotherapy [29], or after four [29] or eight weeks of intervention [30]. It should be noted that there was only one study in each subgroup. In addition, these studies had a small sample size, poor methodological quality [29], and some limitations, such as a high dropout rate [30].

### 4.3. Kinesiophobia

The results showed significant differences in favor of VR when compared with no VR in kinesiophobia postintervention and at the six month followup. When VR + physiotherapy were compared with no VR exercises + physiotherapy, the subgroup analysis showed significant differences in favor of VR in kinesiophobia postintervention and at the six month followup. These studies used Prokin System as the VR intervention, so significant differences in favor of Prokin System also were observed in this subgroup. The intervention lasted 4 weeks in all these articles, thus, the same results were found in subgroups based on duration of intervention. Although these articles had a good methodological quality, they only included young athletic men and their results cannot be generalized [35,36]. The other study that assessed kinesiophobia postintervention, Sato et al. [38] compared Nintendo Switch with oral treatment and the duration of the intervention was 8 weeks. In all of the different subgroups realized significant differences were found in favor of oral treatment. It must be taken into account that there was only this study in each subgroup (Nintendo and 8 weeks).

### 4.4. Virtual Reality in Other Populations

Other studies explored the effects of VR in different populations (such as, patients with chronic neck pain, fibromyalgia, acute pain, Parkinson’s disease, stroke, etc.) and the results differ in part from ours.

In similar chronic pathologies, for example chronic neck pain, VR showed significant differences compared with no VR in pain intensity postintervention, which is in line with our results [39,40]. In this case, interventions consisted of VR compared with physiotherapy and in our review this type of comparison did not obtain significant results. No significant differences were found between VR + physiotherapy versus physiotherapy in pain intensity postintervention [41], which coincides with our review. However, in patients with fibromyalgia, VR combined with exercises showed significant improvement compared to exercises [42,43], although, results in pain intensity are not always conclusive [42]. In addition, in chronic neck pain, significant differences were observed in favor of VR in disability postintervention [39,40] which differs with the results found in our studies. These differences can be explained; the VR interventions in chronic neck pain articles were immersive, and the articles had better methodological quality.

In acute pain pathologies, VR has proven to be an adjuvant tool that can reduce procedural pain [44], burn pain, and anxiety [45]. In addition, it can reduce the use of medication [45]. As we have mentioned previously, in our review the studies that made a comparison between VR + another treatment versus same treatment did not obtain significant differences. In this case, this may be due to differences in the duration of pain and its origin.

Regarding neurological pathologies, such as Parkinson’s disease, VR rehabilitation showed better results in overall improvement than conventional rehabilitation [46]. However, in another study, VR combined with exercises was statistically as effective as each intervention alone [47]. In any case, these results do not agree with ours, since no significant differences were found for these comparisons in CLBP. In stroke patients, VR combined with conventional physical therapy obtained significantly higher improvements than conventional physical therapy [48,49,50]. These results are also not in line with the current review.

### 4.5. Discussion with Other Reviews

The results obtained in our meta-analysis differ partially from those found in other reviews. Bordeleau et al. [16] found significant differences in favor of VR versus no VR interventions for pain intensity postintervention, which is in line with our results. Nevertheless, there are differences in subgroup analysis. When we compared VR with no intervention, the subgroup analysis showed significant differences in favor of VR but in Bordeleau et al. [16] significant differences were not found. The differences found between the meta-analysis may be due to the different articles included in each one and how they was carried out. In addition, they included studies with back pain, whereas we only included studies limited to CLBP patients. In Gumaa et al. [14] the results of the meta-analysis did not show significant differences between VR interventions compared to no intervention in pain intensity postintervention. It should be noted that in one of the studies there was an intervention, since there was electrotherapy [26], and another had a short intervention [51] compared to the others, so we did not consider it comparable. Our results showed significant differences in favor of VR versus no intervention. This can be explained by the greater number of articles included in our meta-analysis and by the different comparisons realized. However, most of the studies included in this meta-analysis had a small sample size, fair methodological quality, and unclear risk of bias.

Bordeleau et al. [16] did not observe significant differences between horse simulator riding and interventions without VR, whereas in our meta-analysis significant differences in favor of horse simulator riding were obtained in pain intensity postintervention. In addition, this is consistent with the results found in two reviews. Collado-Mateo et al. [52] concluded that horse-riding simulators are a promising tool to reduce pain intensity in low back patients, but the interpretation of the results must be performed with extreme caution due to the large heterogeneity, the low number of studies, and the potential risk of bias. Ren et al. [53] also found significant differences in favor of horse simulator riding compared with control in pain intensity postintervention and severity of disability in people with CLBP. However, Ren el al. included another type of VR in addition to horse simulator riding and patients with subacute low back pain.

In Bordeleau et al. [16] the results showed that the potential beneficial effect of VR was more important when more than 12 sessions were performed. In our review, the interventions of included articles lasted 4, 8, or 12 weeks. In all of these cases significant differences in favor of VR were found in pain intensity postintervention, but it should be noted that the best results were obtained in the 12 weeks of intervention subgroup. However, only one study was included [31].

### 4.6. Strengths and Limitations

This review represents an update in the knowledge about the effects of VR treatment in CLBP, incorporating a meta-analysis of outcomes that could not be performed before.

The strengths of the current systematic review included following the PRISMA guidelines [17] for implementation and the use of the PICOS strategy to define the inclusion criteria. Another strength was the performance of meta-analysis. The assessment of methodological quality was carried out with the Downs and Black scale [19], one of the six best scales of methodological quality [21]. Additionally, the risk of bias was assessed with the Cochrane Risk of Bias Assessment Tool [25]. Furthermore, the review was previously registered in PROSPERO with registration number CRD42020222129.

However, although PRISMA guidelines were adhered to and the methodology was strictly followed, completely accounting for the limitations of the included studies was impossible. One of the main limitations was the high heterogeneity between included studies and the difficulties found in making comparable subgroups in order to draw solid conclusions. There were also differences in the age ranges and in the clinical profile of the participants. Regarding the characteristics of the patients, in four studies pain was defined as nonspecific [7,29,30,32], in another study pain was related to work [26]. Four studies [27,35,36,38] made reference to nonspecific pain, however, pain was not defined as nonspecific in the inclusion criteria of these studies. Finally, in three studies pain may have been related to sports practice [34,35,36], as the participants were football players. These differences in the origin of pain must be taken into account, because they could influence the results. Furthermore, the sample size of the included studies was relatively small in some of the studies (19 to 179) and there are no data on long-term outcomes. Finally, three studies compared VR with no intervention and it was expected that results in favor of VR would be observed.

### 4.7. Clinical Implications for Practice

VR interventions could be integrated into clinical practice to reduce pain intensity and kinesiophobia in patients with CLBP, with good results in the short and midterm followup. However, its effects on midterm followup have only been analyzed in a specific population of young sportsmen and cannot be generalized to the general population. Evidence for the efficacy of VR in disability associated with low back pain remains limited. Of the different types of VR, the Prokin System and horse simulator riding have obtained the best short-term results. However, only the studies using the Prokin System showed significant differences at midterm followup (6 months). In addition, this type of VR was combined with physiotherapy. Regarding the duration of the program, an intervention of 12 weeks showed the best results. However, interventions of 4 or 8 weeks also obtained significant results in favor of VR.

### 4.8. Future Research

None of the included studies assessed the variables at long-term followup so future research needs to focus on long-term effects. It may be interesting to conduct more studies comparing VR and physiotherapy versus physiotherapy due to the results obtained in other populations and the low quality of the studies included in this review. Prokin System and horse simulator riding showed good results in the treatment of CLBP. However, these devices are sophisticated and specialized and can be difficult to obtain for a clinic. Therefore, more studies would be necessary to explore the effects of Nintendo consoles in the treatment of CLBP. Although its results are inconclusive, it is commercially available and easier to implement in clinical practice. In addition, there is the possibility of it being used at home. Most of the studies included in this review have been conducted in adult patients under 30 years of age, and our best results were obtained in studies that only included young sportsmen. So, studies of similar quality in other types of populations are needed.

## 5. Conclusions

The results suggest that VR interventions can significantly reduce pain intensity and kinesiophobia in patients with CLBP after the intervention and at the 6 month followup. However, these studies showed high heterogeneity among them, influencing the consistency of the results. VR treatment showed the best results when it was compared with no intervention, placebo, or oral treatment in pain intensity postintervention. VR combined with physiotherapy versus no VR exercise and physiotherapy obtained significant differences in pain intensity and kinesiophobia postintervention and at the six month followup. Regarding VR systems, the Prokin System and horse simulator riding were the most effective short-term. Evidence of Nintendo consoles is still inconclusive, but they present some advantages, so more research is necessary. In terms of the duration of the program, 4, 8, or 12 week interventions showed good results. Studies are needed to evaluate the long-term effects of these interventions.

## Figures and Tables

**Table 2:** Characteristics of interventions.

Author (Year) [Ref.]	Interventions	Session Duration	Frequency	Program Duration	Supervision	Adverse Events
Park et al. (2013) [26]	**EG1: Physical therapy (50 min) + Nintendo Wii exercises (30 min)** *Game*: Nintendo Wii sports. *VR program*: wakeboard, frisbee dog, jet ski, and canoe games. Participants controlled a virtual character on the screen by swinging, rowing, and tilting remote controllers with motion sensors. Participants chose which program they performed. *Time using videogame*: 30 min (2 min break every 10 min).	80 min	3 sessions per week	8 weeks	-	-
	**EG2: Physical therapy (50 min) + Lumbar stabilization exercises (30 min)** 7 positions based on the back bridge, hands and knees, and side bridge. Maintain each position for 15 s for 3 sets.					
	**CG: Physical therapy (50 min)** Hot pack (30 min), interferential current therapy (15 min), and deep heat with ultrasound (5 min).	50 min				
Oh et al. (2014) [27]	**EG1: Horse simulator riding (10 min)** *VR system*: Horse simulator machine (HJL Co. Ltd., Korea). *VR program*: warmup (stretching 5 min + ordinary walking on the horse simulator 5 min) + work-out (sitting trotting and rising trotting 10 min) + cool-down (supine stretching 10 min). *Time using videogame*: 15 min.	30 min	5 sessions per week	8 weeks	Supervised	-
	**EG2: Horse simulator riding (20 min)** *VR system*: Horse simulator machine (HJL Co. Ltd., Korea). *VR program*: warmup (stretching 5 min + ordinary walking on the horse simulator 5 min) + work-out (sitting trotting and rising trotting 20 min) + cool-down (supine stretching 10 min). *Time using videogame*: 25 min.	40 min				
	**EG3: Horse simulator riding (30 min)** *VR system*: Horse simulator machine (HJL Co. Ltd., Korea). *VR program*: warmup (stretching 5 min + ordinary walking on the horse simulator 5 min) + work-out (sitting trotting and rising trotting 30 min) + cool-down (supine stretching 10 min). *Time using videogame*: 35 min.	50 min				
	**CG:** No intervention.	-	-		-	
Yoo et al. (2014) [28]	**EG: Horse simulator riding** *VR system*: Horse simulator machine (HJL Co. Ltd., Korea). *VR program*: warmup (stretching 10 min) + work-out + cool-down (stretching 10 min). *Workout*: ordinary walking and sitting trotting (week 1), increase riding time and riding trotting (weeks 2–3), increase riding time and intensity (weeks 4–5), and increase riding time and intensity (weeks 6–8). *Time using videogame*: 10 min (week 1), 20 min (weeks 2–3), 30 min (weeks 4–5) and 40 min (weeks 6–8).	Week 1: 30 min Week 2–3: 40 min Week 4–5: 50 min Week 6–8: 60 min	3 sessions per week	8 weeks	Supervised	-
	**CG:** No intervention.	-	-		-	
Monteiro-Junior et al. (2015) [32]	**EG: Core exercises and strength training + 8 Wii Fit Plus workout** *VR system*: Nintendo Wii Motion and Wii Balance Board Games. *Games*: chair, tightrope walk, ski slalom, balance bubble, table tilt, sideways, rowing squat, lunge. *VR program*: familiarization, play games 2 times (3 initial sessions). Only one attempt for each game (from 4 session). Verbal stimulus and rest periods (1–2 min). *Time using videogame*: 30 min.	90 min	3 sessions per week	8 weeks	Supervised by a physiotherapist	- Vertigo
	**CG: Core exercises and strength training** Core exercises: postures (15–30 s, 3 series, rest 10–15 s) + Strength training: exercises (10 reps, 3 series).					
Chen et al. (2016) [29]	**EG: Lumbar strengthening exercise (15 min) + Horse simulator riding (15 min)** *VR system:* indoor riding machine (Hongjin Leports, South Korea). *VR program*: the horse simulator riding used in this study simulated riding a real horse through the visual information that appeared on the front screen by the virtual environment. *Time using videogame*: 15 min.	30 min	3 sessions per week	4 weeks	-	-
	**CG: Lumbar strengthening exercise** The exercise program consisted of 6 movements for 1 set. Each movement was held for 5s with 8 reps. All the programs were repeated for 5 times for 1 set.					
Zadro et al. (2019) [7]	**EG: Wii Fit U exercises at home** *VR system*: Nintendo Wii U console and Wii Fit U software. *VR program*: booklet with exercises preselected by the research team (flexibility, strengthening, and aerobic exercises). *Categories*: yoga, muscle, aerobic, and balance. *Intensity*: 13 on the Borg rating scale + symptoms 24 h after exercise. 1 day of rest between exercise sessions. *Learning session*: 1 or 2 h. *Time using videogame*: 60 min.	60 min	3 sessions per week	8 weeks	Unsupervised: EG received fortnightly calls from a physiotherapist	No adverse events were reported
	**CG:** No intervention.	-	-		-	-
Kim et al. (2020) [30]	**EG: Simulated horseback riding** *VR system*: Horse simulator (FORTIS-102; Daewon FORTIS, Republic of Korea). *VR program*: stretching and cooldown (10 min) + workout (30 min) + rest time (6 min). *Workout*: consisted of walking, slow trotting, and fast trotting of a real horse gait. *Time using videogame*: 30 min.	46 min	2 sessions per week	8 weeks	Supervised by practitioner	No adverse events were reported
	**CG: Stabilization exercises** Stretching and cooldown (10 min) + workout (30 min) + rest time (6 min). The stabilization exercises with suspension (Redcord AS, Norway) consisted of a supine pelvic lift, supine and prone bridging exercise, and side-lying hip abduction. Each movement was performed forabout 10 s.					
Nambi et al. (2020) A [34]	**EG1: VR training + Physiotherapy** *VR system*: ProKin system PK252N (Pelvic Module balance trunk MF; TechnoBody, Lanusei, Italy). *Game*: VR balance training with shooting game. The game is controlled by moving the trunk back and forth and left and right according to the signs. The activities were made gradually more difficult with more participant muscle activity and movement. Level of difficulty was defined by the number of enemies, angle of throw, frequency of shoot, frequency of flashing of enemies, and number of balls appearing around the participant. *Time using video game*: 30 min.	30 min + 25 min	5 sessions per week	4 weeks	Supervisor	-
	**EG2: Isokinetic training + Physiotherapy** Warmup: stretching (5 min) + Isokinetic dynamometer: exercise at an angular speed of 60°, 90°, and 120° (15 reps of 3 sets and rest between sets 30 s and between pace 60 s).	− + 25 min				
	**CG: Conventional balance training + Physiotherapy** Active isotonic exercise and isometric exercise (10–15 reps/day) + stretching (3 reps for 10 s).	− + 25 min				
	**Physiotherapy:** Hot pack therapy (20 min) and ultrasound (5 min) + home-based exercise protocol.	25 min				
Nambi et al. (2020) B [35]	**EG1: VR training + Physiotherapy** *VR system*: ProKin system PK252N (Pelvic Module balance trunk MF; TechnoBody, Lanusei, Italy). *Game*: VR balance training with shooting game. The game is controlled by moving the trunk back and forth and left and right according to the signs. The activities were made gradually more difficult with more participant muscle activity and movement. Level of difficulty was defined by the number of enemies, angle of throw, frequency of shoot, frequency of flashing of enemies, and number of balls appearing around the participant. *Time using video game*: 30 min.	30 min + 25 min	5 sessions per week	4 weeks	Supervisor	-
	**EG2: Isokinetic training + Physiotherapy** Warmup: stretching (5 min) + Isokinetic dynamometer: exercise at an angular speed of 60°, 90°, and 120° (15 reps of 3 sets and rest between sets 30 s and between pace 60 s).	− + 25 min				
	**CG: Conventional balance training + Physiotherapy** Active isotonic exercise and isometric exercise (10–15 reps/day) + stretching (3 reps for 10 s).	− + 25 min				
	**Physiotherapy:** Hot pack therapy (20 min) and ultrasound (5 min) + home-based exercise protocol.	25 min				
Park et al. (2020) [31]	**EG: Equine riding simulator** *VR system*: Horse simulator (FORTIS 101, Daewon, Corp., Seoul, Korea). *VR program*: warmup (8 min) + workout (15 min) + cooldown (7 min). *Work-out*: walking (weeks 1–4), walking 10 min + trotting 5 min (weeks 5–8) and trotting 10 min + cantering 5 min (weeks 9–12). *Time using videogame*: 15 min.	30 min	3 sessions per week	12 weeks	Supervised by researcher	-
	**CG: Watching video** Participants sat on the horse and watched the video from the monitor					
Tomruk et al. (2020) [37]	**EG: Computer-based stability training** *VR system*: Biodex Balance System. *VR program*: postural stability training, limits of stability training, weight shift training, and maze control training. 12 stability levels, 3 trials with 10 s rest in each condition. *Time using videogame*: 30 min.	30 min	2 sessions per week	12 weeks	Supervised by physiotherapist	-
	**CG: Traditional training** Traditional postural control exercises by giving them visual, vestibular, or proprioceptive stimulus under the cues of a physiotherapist.					
Garcia et al. (2021) [33]	**EG: EaseVRx at home** *VR system*: Pico G2 4K all-in-one head-mounted VR device. *VR program*: the program delivers a multifaceted combination of pain relief skills training through a prescribed sequence of daily immersive experiences (3D images). Each VR experiences lasts between 2–16 min (average 6 min). *Categories*: pain education, relaxation/interoception, mindful escapes, pain distraction games and dynamic breathing. *Time using video game*: 2–16 min depending on the experience.	2–16 min	7 sessions per week	8 weeks	Unsupervised	- Nausea - Motion sickness
	**CG: Sham VR at home** *VR system*: Pico G2 4K all-in-one head-mounted VR device. *VR program*: sham VR headset displayed 2D nature footage with neutral music that was selected to be neither overly relaxing, aversive, nor distracting. The experience of Sham VR is similar to viewing nature scenes on a large-screen television and is not interactive. Twenty videos were rotated over the 56 sessions, with average duration of sessions closely matching those of EaseVRx.					
Nambi et al. (2021) [36]	**EG1: VR training + Physiotherapy** *VR system*: Pro-Kin system PK 252N (Pelvic Module balance trunk MF; TechnoBody, Lanusei, Italy). *Game*: shooting game. The subjects are sitting on the virtual platform and visualizing the game on the computer display screen. The game was executed and controlled by moving the trunk back and forth and left and right according to the signs. The activities were made gradually more difficult with more participant muscle activity and movement. The level of difficulty was defined by the number of enemies, angle of throw, frequency of shoot, frequency of flashing of enemies, and number of balls appearing around the participant. *Time using videogame*: 30 min.	30 min + 25 min	5 sessions per week	4 weeks	Supervised by physiotherapist	-
	**EG2: Combined physical rehabilitation + Physiotherapy** The participant received balance training through a Swiss ball for core muscles. *Exercises*: wall squat, Russian twist, leg lift, plank saw, cobra and hip raise on the Swiss ball, 15 times per set for 3 sets. Maintain each position 10 s with a 3 s break between repetitions.	− + 25 min				
	**CG: Conventional balance training + Physiotherapy** Active isotonic and isometric exercises for abdominal muscles, deep abdominal muscles, and back muscles. 10 to 15 reps. Stretching was focused on each muscle group for 3 reps for 10 s per muscle group.	− + 25 min				
	**Physiotherapy:** Hot pack therapy (20 min) + ultrasound (5 min) + home-based exercise protocol.	25 min				
Sato et al. (2021) [38]	**EG: Nintendo Ring Fit Adventure Exergame** *VR system*: Ring Fit Adventure of Nintendo Switch. *VR program*: Adventure mode (30 min) + low back pain improvement program (10 min). Ring Fit Adventure is a fitness role-playing game that uses a ring-shaped controller as a device for resistance training. The player advances the story while exercising as the movement of the player is linked to the main character on the screen. *Time using videogame*: 40 min.	40 min	1 session per week	8 weeks	-	-
	**CG: Oral treatment** *Drugs*: Nonsteroidal Anti-Inflammatory Drugs, Tramadol, and Duloxetine. Each drug was started at the standard dose, with patients coming in every 2 weeks to be interviewed for pain, and if pain relief was not adequate, then the dose was gradually increased to its highest recommended level. If pain relief was still inadequate, the next drug was added.	-	-			

EG: Experimental Group; min: minutes; s: seconds; CG: Control Group; VR: Virtual Reality; reps: repetitions.

## Data Availability

The data presented in this study are available in selected articles in the reference list.

## Appendix A

**Table A1:** Search Strategy Studies.

Database	PubMed
Date	05/08/2021
Strategy	#1 AND #2
#1	(“back pain”[Mesh] OR “back pain” OR “low back pain”[Mesh] OR “backache” OR “spine pain” OR “spinal pain” OR “lumbago” OR “sciatica”)
#2	(“Video Games”[Mesh] OR “video game*” OR “videogame*” OR “Gaming” OR “Game” OR “games” OR “Wii” OR “Nintendo” OR “Kinect” OR “Xbox” OR “PlayStation” OR “Virtual Reality”[Mesh] OR “virtual reality” OR “Virtual Reality Exposure Therapy”[Mesh] OR “exergame*” OR “gamification” OR “virtual” OR “computer-based” OR “augmented reality” OR “horse riding” OR “horseback” OR “hippotherapy simulator” OR “equine simulator”)
Database	**Web of Science**
Date	05/08/2021
Strategy	#1 AND #2
#1	TS = (“back pain”[Mesh] OR “back pain” OR “low back pain”[Mesh] OR “backache” OR “spine pain” OR “spinal pain” OR “lumbago” OR “sciatica”)
#2	TS = (“Video Games”[Mesh] OR “video game*” OR “videogame*” OR “Gaming” OR “Game” OR “games” OR “Wii” OR “Nintendo” OR “Kinect” OR “Xbox” OR “PlayStation” OR “Virtual Reality”[Mesh] OR “virtual reality” OR “Virtual Reality Exposure Therapy”[Mesh] OR “exergame*” OR “gamification” OR “virtual” OR “computer-based” OR “augmented reality” OR “horse riding” OR “horseback” OR “hippotherapy simulator” OR “equine simulator”)
Database	**Scopus**
Date	07/08/2021
Strategy	#1 AND #2
#1	TITLE-ABS-KEY (“back pain” OR “low back pain” OR “backache” OR “spine pain” OR “spinal pain” OR “lumbago” OR “sciatica”)
#2	TITLE-ABS-KEY (“video game*” OR “videogame*” OR “Gaming” OR “Game” OR “games” OR “Wii” OR “Nintendo” OR “Kinect” OR “Xbox” OR “PlayStation” OR “virtual reality” OR “Virtual Reality Exposure Therapy” OR “exergame*” OR “gamification” OR “virtual” OR “computer-based” OR “augmented reality” OR “horse riding” OR “horseback” OR “hippotherapy simulator” OR “equine simulator”)
Database	**Cinahl**
Data	06/08/2021
Strategy	#1 AND #2
#1	AB (“back pain”[Mesh] OR “back pain” OR “low back pain”[Mesh] OR “backache” OR “spine pain” OR “spinal pain” OR “lumbago” OR “sciatica”)
#2	AB (“Video Games”[Mesh] OR “video game*” OR “videogame*” OR “Gaming” OR “Game” OR “games” OR “Wii” OR “Nintendo” OR “Kinect” OR “Xbox” OR “PlayStation” OR “Virtual Reality”[Mesh] OR “virtual reality” OR “Virtual Reality Exposure Therapy”[Mesh] OR “exergame*” OR “gamification” OR “virtual” OR “computer-based” OR “augmented reality” OR “horse riding” OR “horseback” OR “hippotherapy simulator” OR “equine simulator”)

## Appendix B

**Table A2:** Search Strategy Ongoing Trials.

Database	ClinicalTrials.gov
Date	17/08/2021
Strategy	(“back pain” OR “low back pain”) AND (“video games” OR “virtual reality” OR “virtual reality exposure therapy”) Filter: study type → interventional (clinical trial)
Database	ISRCTN registry
Date	17/08/2021
Strategy	“back pain” AND “virtual reality” “back pain” AND “virtual reality exposure therapy” “back pain” AND “video games” “low back pain” AND “virtual reality” “low pain” AND “virtual reality exposure therapy” “low back pain” AND “video games”
Database	ICTRP
Date	28/08/2021
Strategy	“back pain” AND “virtual reality” “back pain” AND “virtual reality exposure therapy” “back pain” AND “video games” “low back pain” AND “virtual reality” “low back pain” AND “virtual reality exposure therapy” “low back pain” AND “video games”

## Appendix C

**Table A3:** Excluded Studies in the Last Screening with Reasons for Exclusion (*n* = 44).

Article	Reason for Exclusion
Virtual Environment Rehabilitation for Patients with Motor Neglect Trial (VERMONT): A Single-Center Randomized Controlled Feasibility Trial	No chronic low back pain
Home-Based Balance Training Using the Wii Balance Board	No chronic low back pain
Interactive Sections of an Internet-Based Intervention Increase Empowerment of Chronic Back Pain Patients: Randomized Controlled Trial	No chronic low back pain
Response latencies to postural disturbances when using a virtual reality balance trainer or wobble board in persons with low back pain	No chronic low back pain treatment
Feasibility, Acceptability and Effects of a Home-Based Exercise Program Using a Gerontechnology on Physical Capacities After a Minor Injury in Community-Living Older Adults: A Pilot Study	No chronic low back pain
Effectiveness of Trunk Balance Exercises and Wii Fit TM Balance Exercises in Managing Disability and Pain in Patients with Chronic Low Back Pain	Not randomized trial
Serious Gaming During Multidisciplinary Rehabilitation for Patients With Chronic Pain or Fatigue Symptoms: Mixed Methods Design of a Realist Process Evaluation	Not randomized trial
Examining virtual reality gaming for pain-related fear and disability in chronic low back pain	Meeting abstract
Using Virtual Reality to Treat Chronic Pain: Virtual Graded Exposure for Chronic Low Back Pain and Virtual Walking for Persistent Neuropathic Pain in Spinal Cord Injury	Meeting abstract
Cost effectiveness of virtual reality game versus clinic based mckenzie extension therapy for chronic non specific low back pain	Meeting abstract
Modulating body-image in people with chronic back pain using virtual reality	Meeting abstract
Preliminary Feasibility of a Graded, Locomotor-Enabled, Whole-Body Virtual Reality Intervention for Individuals with Chronic Low Back Pain	Not randomized trial
RabbitRun: An Immersive Virtual Reality Game for Promoting Physical Activities Among People with Low Back Pain dagger	Not randomized trial
Virtual Reality Serious Game for Musculoskeletal Disorder Prevention	Not randomized trial
Exploring the role of pain-related fear and catastrophizing in response to a virtual reality gaming intervention for chronic low back pain	Meeting abstract
Effects of a Nintendo Wii exercise program versus Tai Chi Chuan on standing balance in older adults: a preliminary study	Not randomized trial
A novel, web-enabled multimedia approach, with 3D virtual reality internal and external human body tours, to support low back pain diagnosis	Not randomized trial
Low Back Pain Attenuation Employing Virtual Reality Physiotherapy	Not randomized trial
Mindfulness-based cognitive-behavior therapy (MCBT) versus virtual reality (VR) enhanced CBT, versus treatment as usual for chronic back pain. A clinical trial	Not randomized trial
A Portable Wireless Solution for Back Pain Telemonitoring: A 3D-Based, Virtual Reality Approach	Not randomized trial
Assessing the Perception of Trunk Movements in Military Personnel with Chronic Non-Specific Low Back Pain Using a Virtual Mirror	Not randomized trial
ALFRED Back Trainer: Conceptualization of a Serious Game-Based Training System for Low Back Pain Rehabilitation Exercises	Not randomized trial
A Virtual Reality Lower-Back Pain Rehabilitation Approach: System Design and User Acceptance Analysis	Not randomized trial
Efficacy of virtual reality to reduce chronic low back pain: Proof-of-concept of a non-pharmacological approach on pain, quality of life, neuropsychological and functional outcome	Not randomized trial
Proposed Game for Promoting Physical Activities among People with Low Back Pain using Virtual Reality	Not randomized trial
The influence of a biopsychosocial educational internet-based intervention on pain, dysfunction, quality of life, and pain cognition in chronic low back pain patients in primary care: a mixed methods approach	Not randomized trial
Tailored, multimedia versus traditional educational interventions for patients with low back pain: a randomized clinical trial.	No virtual reality intervention
Seeing It Helps: Movement-related Back Pain Is Reduced by Visualization of the Back During Movement	No virtual reality intervention
New exercise system for waist and back and its effect detection	No virtual reality intervention
Tele-rehabilitation for back pain in Korean farmers	No virtual reality intervention
Body schema acuity training and Feldenkrais RTM movements compared to core stabilization biofeedback and motor control exercises: Comparative effects on chronic non-specific low back pain in an outpatient clinical setting: A randomized controlled comparative study	No virtual reality intervention
Effect of Motor Control Training on Muscle Size and Football Games Missed from Injury	No virtual reality intervention
Randomized trial comparing interferential therapy with motorized lumbar traction and massage in the management of low back pain in a primary care setting	No virtual reality intervention
Self-Administered Skills-Based Virtual Reality Intervention for Chronic Pain: Randomized Controlled Pilot Study	Not only spinal pain
Effects of physiotherapy associated to virtual games in pain perception and heart rate variability in cases of low back pain	No chronic low back pain
Adherence to home exercises in non-specific low back pain. A randomised controlled pilot trial.	No chronic low back pain
Radiological (Magnetic Resonance Image and Ultrasound) and biochemical effects of virtual reality training on balance training in football players with chronic low back pain: A randomized controlled study.	No variables related to pain
The Effects of VR-based Wii Fit Yoga on PhysicalFunction in Middle-aged Female LBP Patients	No chronic low back pain
Evaluation of biofeedback based bridging exercises on older adults with low back pain: A randomized controlled trial	No chronic low back pain
Is physiotherapy integrated virtual walking effective on pain, function, and kinesiophobia in patients with non-specific low-back pain? Randomied controlled trial	No chronic low back pain
Effect of hippotherapy simulator on pain, disability and range of motion of the spinal column in subjects with mechanical low back pain: A randomized single-blind clinical trial	No chronic low back pain
A change in the size of the abdominal muscles and balance ability after virtual reality exercise in the elderly with chronic low back pain	No variables related to pain
Feasibility and Safety of a Virtual Reality Dodgeball Intervention for Chronic Low Back Pain: A Randomized Clinical Trial	Intervention duration < 4 weeks
Virtual reality distraction induces hypoalgesia in patients with chronic low back pain: a randomized controlled trial	Intervention duration < 4 weeks

## Appendix D

**Table A4:** Characteristics of Included Registry Entries or Ongoing Trials (*n* = 17).

Number	Article	Recruitment Status
NCT02125968	Therapeutic Effects of Video Game Play Therapy on Patients With Chronic Low Back Pain	Unknown status
NCT03819907	The Use of Virtual Reality for Lumbar Pain Management in an Outpatient Spine Clinic	Completed
NCT04468074	Virtual Reality Treatment for Adults With Chronic Back Pain	Completed
NCT04042090	The Efficacy, Acceptability, Tolerability and Feasibility of a Therapeutic Virtual Reality Application	Active, not recruiting
NCT04273919	Virtual Reality for the Treatment of Chronic Low Back Pain	Recruiting
NCT04236804	Implementing TMC-CP01 Treatment Based on the Virtual Autonomic Neuromodulation Induced Systemic Healing System in Reducing Pain and Opioid Requirement in Subjects Suffering From Chronic Low Back Pain	Recruiting
NCT04307446	Immersive Virtual Reality and Chronic Back Pain	Recruiting
NCT04139564	EaseVRx for the Reduction of Chronic Pain and Opioid Use	Recruiting
NCT04225884	Digital Therapeutics (DTx) for Pain: Pilot Study of a Virtual Reality Software for Chronic Pain	Recruiting
NCT04609787	Immersive Virtual Reality and Central Sensitization in People With Chronic Pain	Recruiting
NCT03909048	Chronic Low Back Pain Graded - Exposure Psychoeducation Intervention	Completed
PACTR202010569932287	Comparative effects of augmented, virtual and mixed reality on pain characteristics and health-related quality of life of patients with chronic non-specific low-back pain.	Not recruiting
PACTR202007533977502	Comparative effects of clinic and virtual reality-based McKenzie extension therapy in chronic non-specific low-back pain.	Not recruiting
IRCT20200330046895N1	Effects of virtual reality exercises on clinical outcomes in patients with chronic low back pain: a randomized controlled trial.	Recruiting
ACTRN12619001776190	Altering body image in chronic low back pain using virtual reality: A proof of concept randomised clinical trial.	Not recruiting
PACTR201907749053096	Effects of Core Stability Exercise Combined with Virtual Reality in Collegiate Athletes with Nonspecific Low Back Pain: A Randomized Clinical Trial.	Not recruiting
JPRN-UMIN000035505	Effect of exercise by Virtual Reality in patients with chronic low back pain.	Not recruiting

## Appendix E

**Table A5:** Methodological Quality of Included Studies.

	Study Quality										External Validity			Study Bias							Confounding and Selection Bias						Study Power		
Author (Year) [Ref.]	1	2	3	4	5	6	7	8	9	10	11	12	13	14	15	16	17	18	19	20	21	22	23	24	25	26	27	Total	Quality
Park et al. (2013) [26]	1	1	1	1	2	1	1	0	1	0	0	0	0	0	0	1	1	1	1	1	1	0	1	0	0	0	0	16	FAIR
Oh et al. (2014) [27]	1	1	1	1	2	1	0	0	0	1	1	0	0	0	0	1	1	1	0	1	1	0	1	0	0	0	0	15	FAIR
Yoo et al. (2014) [28]	1	1	1	1	2	1	0	0	1	1	1	0	0	0	0	1	1	0	1	1	1	0	1	0	0	1	0	17	FAIR
Monteiro-Junior et al. (2015) [32]	1	1	1	1	1	1	1	1	1	0	0	0	1	0	1	1	1	1	1	1	1	0	1	1	0	1	1	21	GOOD
Chen et al. (2016) [29]	1	1	1	1	0	0	1	0	0	1	0	0	0	0	0	1	1	1	0	1	1	0	1	0	0	0	1	13	POOR
Zadro et al. (2019) [7]	1	1	1	1	1	1	1	1	1	1	1	1	1	0	1	1	1	1	1	1	1	1	1	1	1	1	1	26	EXCELLENT
Kim et al. (2020) [30]	1	1	1	1	0	1	1	1	1	1	1	1	1	0	0	1	1	1	1	1	1	1	1	0	1	0	1	22	GOOD
Nambi et al. A (2020) [34]	1	1	1	1	2	1	1	0	1	1	1	1	0	1	1	1	1	1	1	1	1	0	1	1	1	1	1	25	GOOD
Nambi et al. B (2020) [35]	1	1	1	1	2	1	1	0	1	1	1	1	0	1	1	1	1	1	0	1	1	0	1	1	1	1	1	24	GOOD
Park et al. (2020) [31]	1	1	1	1	2	1	1	0	1	1	0	1	0	1	0	1	1	1	1	1	0	0	1	0	1	1	1	21	GOOD
Tomruk et al. (2020) [37]	1	1	1	1	2	1	1	0	1	1	0	0	0	0	0	1	1	1	1	1	1	0	1	0	0	0	1	18	FAIR
Garcia et al. (2021) [33]	1	1	1	1	2	1	1	1	1	1	1	1	1	1	0	1	1	1	1	1	1	1	1	1	1	1	1	27	EXCELLENT
Nambi et al. (2021) [36]	1	1	1	1	2	1	1	0	1	1	0	1	0	1	1	1	1	1	1	1	1	0	1	1	1	1	1	24	GOOD
Sato et al. (2021) [38]	1	1	1	1	2	1	1	0	1	1	1	0	1	0	0	1	1	1	1	1	1	1	1	0	0	1	1	22	GOOD
